# Computation of contrast-enhanced perfusion using only two CT scan phases: a proof-of-concept study on abdominal organs

**DOI:** 10.1186/s41747-022-00292-y

**Published:** 2022-08-29

**Authors:** Massimo Cressoni, Andrea Cozzi, Simone Schiaffino, Paolo Cadringher, Paolo Vitali, Gianpaolo Basso, Davide Ippolito, Francesco Sardanelli

**Affiliations:** 1 Unit of Radiology, IRCCS Policlinico San Donato, Via Morandi 30, 20097 San Donato Milanese, Italy; 2 Elekton S.A.S., Regione Crena 15A, 14041 Agliano Terme, Italy; 3 Department of Biomedical Sciences for Health, Università degli Studi di Milano, Via Mangiagalli 31, 20133 Milan, Italy; 4 School of Medicine and Surgery, Università degli Studi di Milano-Bicocca, Piazza dell’Ateneo Nuovo 1, 20126 Milan, Italy; 5 Department of Radiology, ASST Monza—Ospedale San Gerardo, Via Pergolesi 33, 20900 Monza, Italy

**Keywords:** Tomography (x-ray computed), Contrast media, Perfusion imaging, Carcinoma (hepatocellular)

## Abstract

**Background:**

Computed tomography perfusion imaging (CTPI) by repeated scanning has clinical relevance but implies relatively high radiation exposure. We present a method to measure perfusion from two CT scan phases only, considering tissue enhancement, feeding vessel (aortic) peak enhancement, and bolus shape.

**Methods:**

CTPI scans (each with 40 frames acquired every 1.5 s) of 11 patients with advanced hepatocellular carcinoma (HCC) enrolled between 2012 and 2016 were retrospectively analysed (aged 69 ± 9 years, 8/11 males). Perfusion was defined as the maximal slope of the time-enhancement curve divided by the peak enhancement of the feeding vessel (aorta). Perfusion was computed two times, first using the maximum slope derived from all data points and then using the peak tissue enhancement and the bolus shape obtained from the aortic curve.

**Results:**

Perfusion values from the two methods were linearly related (*r*^2^ = 0.92, *p* < 0.001; Bland–Altman analysis bias -0.12). The mathematical model showed that the perfusion ratio of two ROIs with the same feeding vessel (aorta) corresponds to their peak enhancement ratio (*r*^2^ = 0.55, *p* < 0.001; Bland–Altman analysis bias -0.68). The relationship between perfusion and tissue enhancement is predicted to be linear in the clinical range of interest, being only function of perfusion, peak feeding vessel enhancement, and bolus shape.

**Conclusions:**

This proof-of-concept study showed that perfusion values of HCC, kidney, and pancreas could be computed using enhancement measured only with two CT scan phases, if aortic peak enhancement and bolus shape are known.

**Supplementary Information:**

The online version contains supplementary material available at 10.1186/s41747-022-00292-y.

## Key points

- Abdominal perfusion can be computed from peak tissue enhancement.
- Perfusion ratio between parenchymal ROIs corresponds to the ratio between their enhancements.
- Perfusion can be estimated from just two CT scans and bolus shape.

## Background

The measurement of organ perfusion carries a scientific and clinical relevance in many fields, such as the evaluation of the response of solid tumours to chemotherapy [1], including anti-angiogenic therapy in hepatocellular carcinoma (HCC) [2, 3]; the differentiation between ischemic penumbra and infarct core in stroke [4], the diagnosis of cerebral vasospasm [5], and the assessment of myocardial ischemia [6].

Current methods to compute perfusion, proposed and implemented between the late 1980s and the early 1990s [7–9], rely on the repeated scanning of a given volume during bolus transit. However, spatial resolution is limited both by the practical impossibility of achieving complete co-registration for all volumes in the series and by the need of curtailing the already high radiation dose implied by this method [10].

The maximum slope method considers a series of CT acquisition of a given volume to calculate perfusion by determining the upslope of the tissue time-enhancement curve and dividing it by the peak enhancement of the feeding vessel: the vessel usually considered for the determination of abdominal perfusion is the aorta [8, 9]. We investigated if it is possible to compute perfusion using only two CT scan data points together with aortic bolus shape and aortic peak enhancement values, retrospectively validating this method on abdominal perfusion CT scans. This approach would allow perfusion computation with lower radiation doses and higher signal-to-noise ratio (SNR).

## Methods

### Patient population

We retrospectively analysed patients from a previously published study on the effects of anti-angiogenic therapy on the vascularity of HCC [3]. The study was approved by the Ethics Committee of ASST Monza–Ospedale San Gerardo (Monza, Italy) and all patients provided written informed consent.

Patients were enrolled between March 2012 and October 2016. Inclusion criteria were (1) a diagnosis of HCC; (2) Child–Pugh class A; (3) Eastern Cooperative Oncology Group performance status 0–1; (4) not having received previous systemic treatment for HCC; (5) no contraindications to CT imaging. Exclusion criteria were (1) Child–Pugh class B and C; (2) previous administration of *c-met* inhibitors; (3) concomitant radiotherapy; (4) a history of other malignancies or their concomitant presence; (5) presence of esophageal varices bleeding or of coagulation disorders; (6) glomerular filtration rate below 30 mL/min.

### CT protocol and scanning parameters

Patients underwent a clinical CT study before and after weight-tailored intravenous administration of an iodinated contrast agent at a 4.5 mL/s flow rate, using a 18 gauge catheter positioned into an antecubital vein. Arterial, portal venous, and equilibrium phases were acquired with a 2 mm collimation (pitch of 0.83). The bolus tracking technique was used to set individual acquisition times for the dynamic phases (*i.e.*, arterial, portal venous, and delayed phases). Images from this part of the original study were not used for the analysis reported in the present manuscript.

To avoid influence of previously administered contrast agent, the perfusion CT study was performed about 45 min afterwards. Perfusion studies were performed as follows: a 50 mL bolus of iodinated contrast (Xenetix 350; Guerbet, Aulnay, France) with a 350 mgI/mL concentration was injected at a 5 mL/s flow rate, acquiring 40 CT scan volumes on a 256-slice multi-detector-row (slab thickness 80 mm) at time intervals of 1.5 s. All patients were imaged on the same 256-slice CT scanner (Brilliance, iCT, Philips Medical Systems, Eindhoven, The Netherlands). Imaging parameters were: 100 kVp, 100 mAs, 512 × 512 matrix, slice thickness 2.5 mm, acquisition time 1.4 s. The acquisition began after a 5 s delay from intravenous contrast agent injection. A strap compressing the abdomen and limiting respiratory excursions was used to reduce respiratory motion artifacts.

### Image analysis

**Fig. 1:**
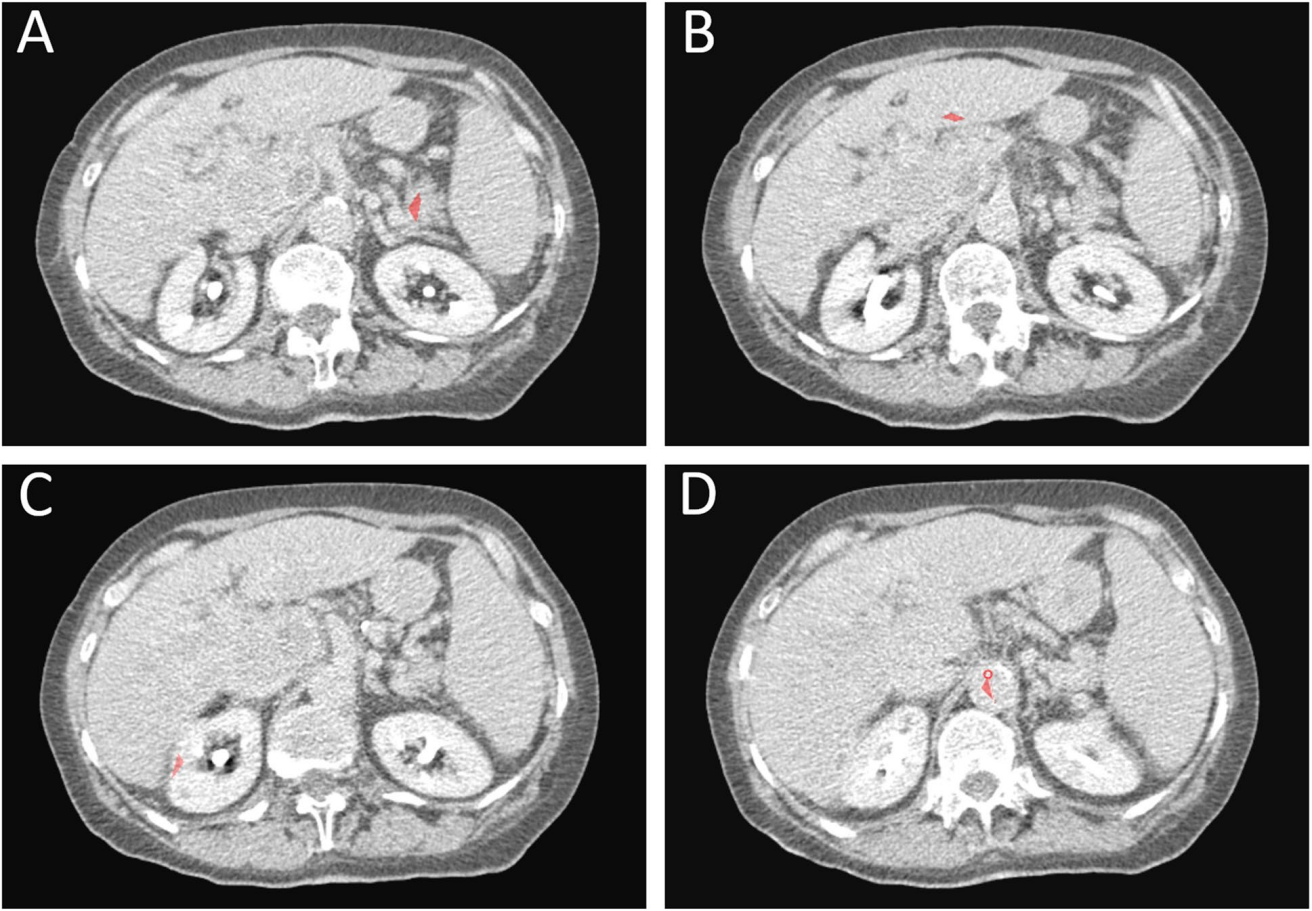
ROIs drawn to compute the time-enhancement curves. In **A**, a ROI on the pancreas was drawn on the largest pancreatic portion included in the CT study volume. While this portion is usually represented by the pancreatic head, in this case the pancreatic head was not included in the study volume due to a pathologic lymph node and the ROI was drawn on the pancreatic tail. **B** ROI drawn on an HCC focus. **C** ROI drawn on the cortex of the right kidney. **D** ROI drawn in the aorta, between the emergency of the superior mesenteric artery and of the renal arteries

Image analysis was performed with a custom-made software (www.softefilm.eu). A trained radiologist (M.C., with 6 years of experience in abdominal imaging) pre-processed perfusion images by drawing four ROIs. The first ROI was drawn on the abdominal aorta between the emergence of the superior mesenteric artery and of the renal arteries: as in the reference study and the original manuscript by Miles [9], this ROI was used to define the feeding vessel time-enhancement curve, assuming that the same curve is conserved in the branching vessels. The second ROI was drawn on a HCC focus without evident necrosis, first selected on the clinical CT scans—because of their higher SNR and of the availability of a delayed acquisition—and afterwards identified and segmented on the perfusion CT images. The last two ROIs were drawn on the cortex of right kidney and on the biggest pancreatic region included in the study volume visible (as exemplified in Fig. 1). Afterwards, the aforementioned custom-made software translated each ROI on the same spatial coordinates of the remaining 39 frames. No attempt was made to spatially register the frames: this fact, combined with image noise, engendered a reduction of the whole quality of time-enhancement curves.

For image quality analysis, we computed the SNR as mean of the pre-contrast aortic HU value divided by the ROI standard deviation. To compute contrast-to-noise ratio (CNR), a fifth ROI was drawn on the paravertebral musculature in order to obtain:

$$\mathrm{CNR}=\frac{\mathrm{Aortic\;mean\;HU\;}-\mathrm{\;muscle\;mean\;HU}}{\mathrm{Muscle\;HU\;standard\;deviation}}$$

For each ROI, mean HU values with their standard deviation were computed alongside median HU values with their interquartile range. Then, the median HU values were used to compute the time-enhancement curve in the abdominal aorta with the Madsen formulation of the gamma variate function [11], as described below, obtaining the peak and α values. Conversely, for ROIs on HCC, kidney cortex, and pancreas, we computed time-enhancement curves from ROI median values with two different methods: (i) using a spline function (in order to remove image noise and obtain smoothed curves); (ii) by fitting a gamma variate function according to the Madsen formulation (to obtain peak tissue enhancement and *t*_max_) [11].

### Perfusion evaluation

Using the whole set of time-enhancement data, the maximum slope method was used to computed perfusion two times: (i) using maximum slope values obtained from the spline function; (ii) using maximum slope values of the function determined as the maximal value of the first derivative of the gamma variate equation (using peak tissue enhancement, *t*_max_, and aortic α value).

### Relationship between perfusion ratios and enhancement ratios

We computed for each patient the ratio between kidney/pancreas, kidney/HCC and pancreas/HCC perfusion and peak tissue enhancements. Perfusion data refer to the perfusions computed as maximal upslope of the time-enhancement curve obtained using all data points smoothed with the spline function.

### Mathematical model

Given that arterial circulation is made of terminal branching vessels, and all vessels in a definite anatomical region have the same circulation time, perfusion $\left(\frac{\displaystyle\frac{ml}s}{ml_{tissue}}\right)$ can be measured as proposed by Peters et al. [8] and Miles [9]:

$$\mathrm{Perfusion}=\frac{\mathrm{Maximum\;slope\;of\;tissue\;enhancement}}{\mathrm{Peak\;feeding\;vessel\;enhancement}}$$

As previously mentioned, we aim to compute the maximum slope of tissue enhancement using only two single-energy CT scans (baseline and peak). Peak feeding vessel enhancement in the clinical setting can be inferred using the bolus test technique [12].

Vessel and tissue enhancement after the injection of a contrast bolus can be described with a gamma variate function [11, 13]. We use here the simplified formulation proposed by Madsen [11]:

$\mathrm{Enhancement}={y}_{\mathrm{max}}\times {t}^{\alpha }\times {e}^{\alpha \left(1-t\right)}$ where *y*_max_ represents the maximum enhancement, *t* is defined as $\mathrm{time}\text{/}{t}_{\mathrm{max}}$ (t_max_ being the time at which the function reaches its maximum), and α describes the bolus shape.

The bolus shape is conserved between arterial input and tissue time-enhancement curves [8, 9]: this allows to determine bolus shape from the arterial input curve (α) and use it together with the measured peak tissue enhancement to compute the tissue time-enhancement curve.

The maximal slope of the curve can be defined as the value of the first derivative of the Madsen equation at the time where the second derivative is 0. The first derivative is

$$dy={y}_{\mathrm{max}}\times \left(\left(\alpha \times {t}^{\alpha -1}\times {e}^{\alpha \left(1-t\right)}\right)-\left(\alpha \times {t}^{\alpha }\times {e}^{\alpha \left(1-t\right)}\right)\right)$$

the second derivative is

$$ddy={y}_{\mathrm{max}}\times \alpha \times {e}^{\alpha \left(1-t\right)}\times {t}^{\alpha -2}\times \left(\alpha \times {t}^{2}-2\times \alpha \times t+\alpha -1\right)$$

and the *t* at which the 0 of the second derivate is

$$t=\frac{\left(\alpha \pm \sqrt{\alpha }\right)}{\alpha }$$

Consequently, we can define a parameter *k* so that:

$$k=\left(\left(\alpha \times {t}^{\alpha -1}\times {e}^{\alpha \left(1-t\right)}\right)-\left(\alpha \times {t}^{\alpha }\times {e}^{\alpha \left(1-t\right)}\right)\right)$$ with $$t=\frac{\left(\alpha \pm \sqrt{\alpha }\right)}{\alpha }$$

and finally:

$$\mathrm{Maximum\;slope\;of\;tissue\;enhancement}={y}_{\mathrm{max}}\times \frac{k}{{t}_{\mathrm{max}}}$$

### Relative perfusions

Considering two different regions of interest (ROIs) we can re-write:

$$\frac{{\mathrm{Organ\;blood\;flow}}_{{\mathrm{ROI}}_{1}}}{{\mathrm{Organ\;blood\;flow}}_{{\mathrm{ROI}}_{2}}}=\frac{\frac{{\mathrm{Maximum\;slope\;of\;tissue\;enhancement}}_{{\mathrm{ROI}}_{1}}}{{\mathrm{Peak\;feeding\;vessel\;enhancement}}_{{\mathrm{ROI}}_{1}}}}{\frac{{\mathrm{Maximum\;slope\;of\;tissue\;enhancement}}_{{\mathrm{ROI}}_{2}}}{{\mathrm{Peak\;feeding\;vessel\;enhancement}}_{{\mathrm{ROI}}_{2}}}}$$

Mathematically, the peak enhancement of the feeding vessel can be removed as:

$$\frac{{\mathrm{Organ\;blood\;flow}}_{{\mathrm{ROI}}_{1}}}{{\mathrm{Organ\;blood\;flow}}_{{\mathrm{ROI}}_{2}}}=\frac{{\mathrm{Maximum\;slope\;of\;tissue\;enhancement}}_{{\mathrm{ROI}}_{1}}}{{\mathrm{Maximum\;slope\;of\;tissue\;enhancement}}_{{\mathrm{ROI}}_{2}}}$$

Then, since

$$\frac{{\mathrm{Maximum\;slope\;of\;tissue\;enhancement}}_{{\mathrm{ROI}}_{1}}}{{\mathrm{Maximum\;slope\;of\;tissue\;enhancement}}_{{\mathrm{ROI}}_{2}}}=\frac{{y}_{{\mathrm{max}}_{\mathrm{ROI}1}}\times \frac{k}{{t}_{\mathrm{max}}}}{{y}_{{\mathrm{max}}_{\mathrm{ROI}2}}\times \frac{k}{{t}_{\mathrm{max}}}}$$

where

$$k=\left(\left(\alpha \times {t}^{\alpha -1}\times {e}^{\alpha \left(1-t\right)}\right)-\left(\alpha \times {t}^{\alpha }\times {e}^{\alpha \left(1-t\right)}\right)\right)$$

with $t=\frac{\left(\alpha \pm \sqrt{\alpha }\right)}{\alpha }$, we can define

$$\frac{{\mathrm{Organ\;blood\;flow}}_{{\mathrm{ROI}}_{1}}}{{\mathrm{Organ\;blood\;flow}}_{{\mathrm{ROI}}_{2}}}=\frac{{y}_{{\mathrm{max}}_{{\mathrm{ROI}}_{1}}}}{{y}_{{\mathrm{max}}_{{\mathrm{ROI}}_{2}}}}$$

Consequently, the ratio of arterial phase tissue enhancement corresponds to the ratio of arterial perfusions.

### Statistical analysis

The relationship between the two perfusion computation methods and the relationship between perfusion ratios and enhancement ratios were investigated with linear regression and Bland–Altman analysis.

## Results

### Patient population

A total of 43 patients were enrolled in the original study. Among them, we retrieved all those for whom the pancreas and right kidney were included in the imaged volume of the pre-treatment CT perfusion studies. Therefore, the population of this study comprised pre-treatment perfusion CT scans of 11 patients (aged 69 ± 9 years, 8/11 males).

### Image quality analysis and patient-based perfusion evaluation

**Fig. 2:**
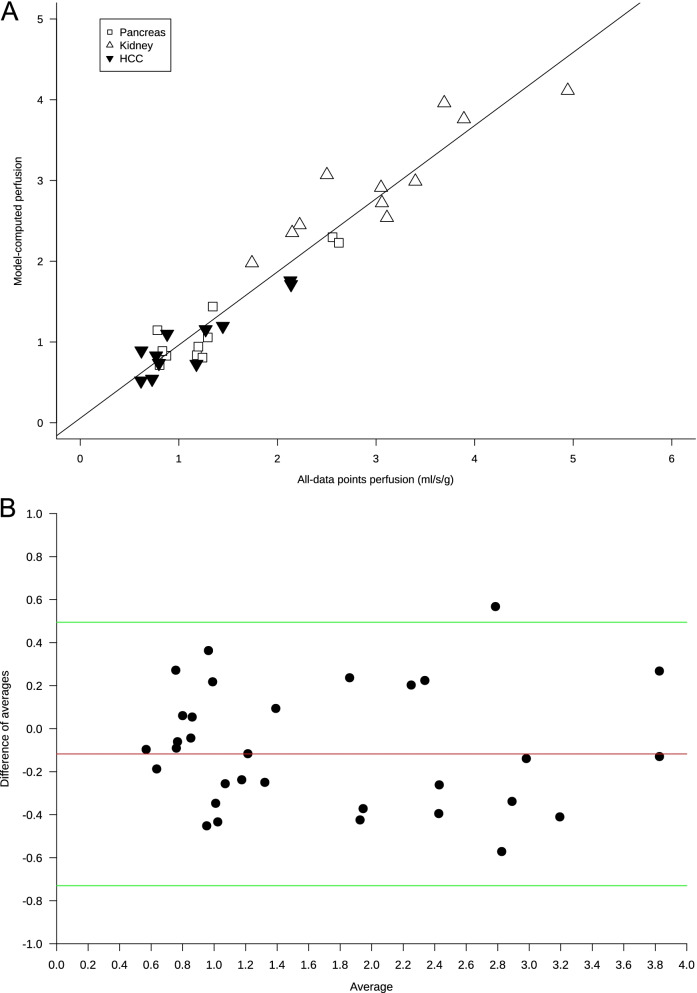
**A** Scatterplot and linear regression of the relationship between the two methods of perfusion measurement. Values on the *x*-axis represent organ perfusion computed using the maximal upslope of the time-enhancement curve using all data points smoothed with a spline function. Values on the *y*-axis indicate organ perfusion interpolated using the maximal upslope of the gamma variate function using peak tissue enhancement and time-enhancement curve shape parameter (α) computed from the aortic curve. Regression equation: $\mathrm{gamma}\;\mathrm{variate}\;\mathrm{based}\;\mathrm{perfusion}\;\left(\frac{\displaystyle\frac{\mathrm{ml}}\min}{\mathrm g}\right)=0.06+0.91\times\left(\mathrm{spline}\;\mathrm{based}\;\mathrm{perfusion}\right)$, *r*^2^ = 0.92, *p* < 0.001. Three points for each patient are included: empty squares for the pancreas, empty triangles for the kidney, and black upside-down triangles for hepatocellular carcinoma (HCC). **B** Bland–Altman plot for the comparison of the two perfusion computation methods. The red line indicates the bias (-0.12), while green lines indicate the lower (-0.73) and upper (0.49) limit of agreement. Only one outlier was observed

**Fig. 3:**
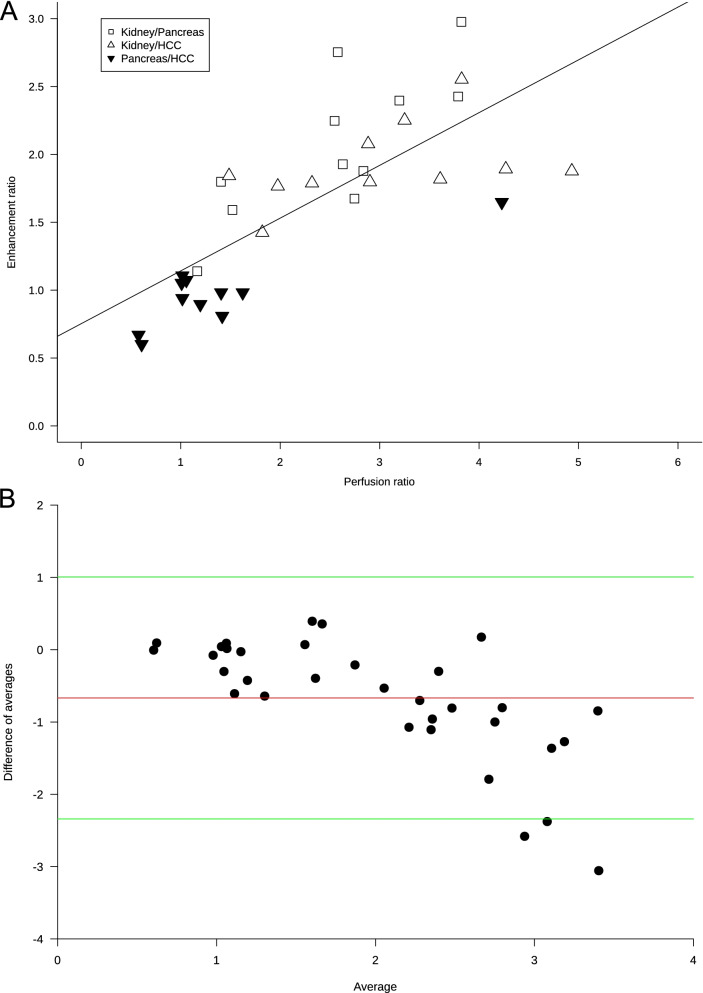
**A** Scatterplot and linear regression of the relationship between ratio of perfusions and peak enhancement ratios. Three points for each patient are included: empty squares for kidney/pancreas data, empty triangles for kidney/hepatocellular carcinoma (HCC) data, and black upside-down triangles for pancreas/HCC data. Regression equation: $\mathrm{enhancement\;ratio}=0.75+0.39\times \left(\mathrm{perfusion\;ratio}\right)$, *r*^2^ = 0.55, *p* < 0.001. Perfusion data refer to the perfusions computed as maximal upslope of the time-enhancement curve obtained using all data points smoothed with the spline function. **B** Bland–Altman plot for the comparison between enhancement and perfusion ratios, showing a -0.68 bias (red line), with green lines indicating the lower (-2.34) and upper (1.01) limit of agreement

Image quality analysis showed a SNR of 2.2 ± 0.73 and a CNR of 0.9 ± 0.59. Perfusion values computed with the two methods were highly related ($\mathrm{gamma}\;\mathrm{variate}\;\mathrm{based}\;\mathrm{perfusion}=0.06+0.091\times\left(\mathrm{spline}\;\mathrm{based}\;\mathrm{perfusion}\right)$, *r*^2^ = 0.92, *p* < 0.001, Fig. 2A), as were the perfusion ratio and the enhancement ratio ($\mathrm{enhancement\;ratio}=0.75+0.39\times \left(\mathrm{perfusion\;ratio}\right)$, *r*^2^ = 0.55, *p* < 0.001, Fig. 3A). Bland–Altman analysis for the comparison of the two perfusion computation methods showed a bias of -0.12 (lower limit of agreement -0.73, upper limit of agreement 0.49) with only one outlier (Fig. 2B), while for the comparison between enhancement and perfusion ratios (Fig. 3B), we found a bias of -0.68 (lower limit of agreement -2.34, upper limit of agreement 1.01).

### Relationship between arterial phase tissue enhancement and perfusion

**Fig. 4:**
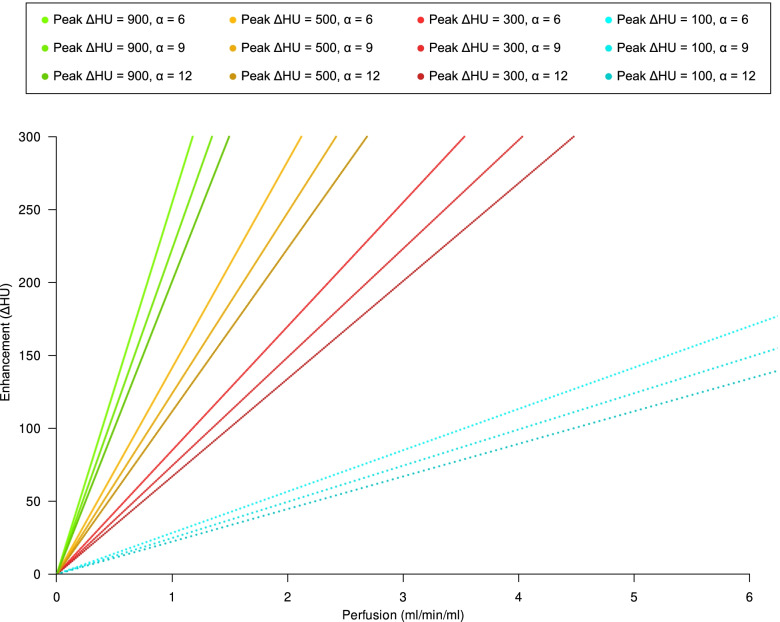
Relationship between peak arterial phase tissue enhancement (peak ΔHU) and organ perfusion (mL/s/mL) in a parenchymatous organ with a *t*_max_ of approximately 35 s for different peak feeding vessel enhancements and α parameters. To estimate peak arterial phase tissue enhancement, we assumed that contrast medium, injected in a peripheral vein or in a central venous catheter, is diluted by the right and left heart into the whole cardiac output before entering arterial circulation. Assuming a negligible total volume change due to contrast agent injection, if we inject 1.25 mL/kg of a iodinated contrast agent with a 350 mgI/mL concentration at 3.2 mL/s flow rate in a 80 kg man with an average cardiac output of 5000 mL/min (approximately 83 mL/s) and we acquire a CT scan at 120 kVp (with a constant of 26.18 for ΔHU calculation), peak ΔHU will then be $\frac{3.2\times 350\times 26.18}{83}=350\;\mathrm{HU}$

**Fig. 5:**
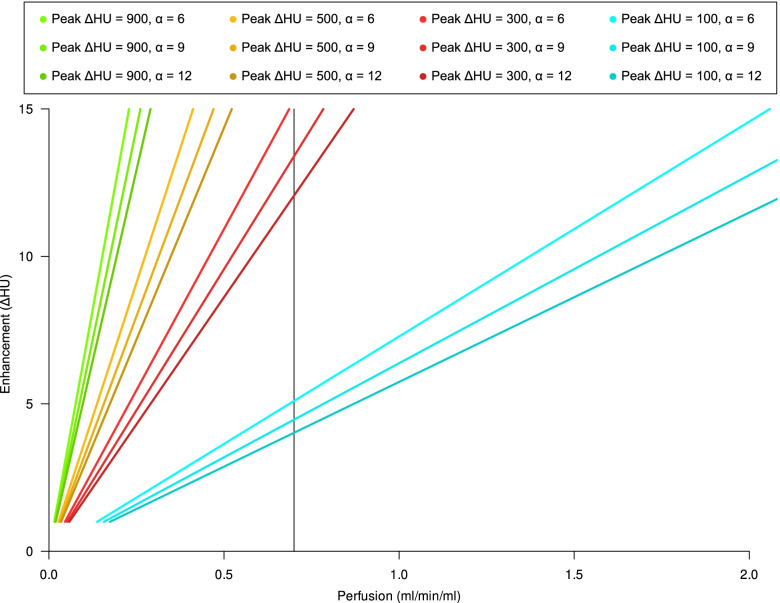
Relationship between peak arterial phase tissue enhancement (ΔHU) and organ perfusion (mL/s/mL) in the brain for different peak feeding vessel enhancements and α parameters. Due to the effect of the blood–brain barrier and to the lack of an interstitial phase, we have a very short *t*_max_ (approximately 9 s). The average perfusion value for the grey matter (indicated by the black vertical line) would be 0.72 mL/min/mL (range 0.64–0.84 mL/min/mL)

The relationship between organ perfusion and arterial phase tissue enhancement is presented in Fig. 4. This relationship intersects the axes origin (no enhancement without perfusion) and its slope depends on the enhancement peak in the feeding vessel and, as a minor contributor, on the steepness of bolus shape, described by the α parameter, as shown in Fig. 3. As an example, in a patient injected with 3.2 mL/s of iodinated contrast agent with a 350 mgI/mL concentration, assuming a normal cardiac output (5 L/min) and image acquisition at 120 kVp, we expect an enhancement of approximately 100 HU in the pancreas with a perfusion of 1.15 mL/min/mL of tissue, and a 220 HU enhancement of the renal cortex with a perfusion of 2.5 mL/min [9]. Figure 5 presents an example of the relationship between brain peak enhancement and perfusion. To a peak feeding vessel enhancement of about 500 HU will correspond a cortical enhancement ranging 10–15 HU.

### Relationship between absolute perfusion and arterial phase tissue enhancement

Perfusion is computed as

$$\frac{{y}_{\mathrm{max}}\times \frac{k}{{t}_{\mathrm{max}}}}{\mathrm{Peak\;feeding\;vessel\;enhancement}}$$

This shows that perfusion is directly proportional to *y*_max_, proportional to α (k is a function of α) and inversely proportional to peak feeding vessel enhancement.

The equation can also be rearranged as ${y}_{\mathrm{max}}=\frac{\mathrm{Perfusion\;}\times \mathrm{\;Peak\;feeding\;vessel\;enhancement}}{\frac{k}{{t}_{\mathrm{max}}}}$ to obtain the tissue enhancement for a given perfusion with a given arterial bolus defined its peak and shape (α).

As mentioned before, the value of *k* can be defined only in term of α as

$$k=\left(\left(\alpha \times {t}^{\alpha -1}\times {e}^{\alpha \left(1-t\right)}\right)-\left(\alpha \times {t}^{\alpha }\times {e}^{\alpha \left(1-t\right)}\right)\right)$$ with $$t=\frac{\left(\alpha \pm \sqrt{\alpha }\right)}{\alpha }$$  

**Fig. 6:**
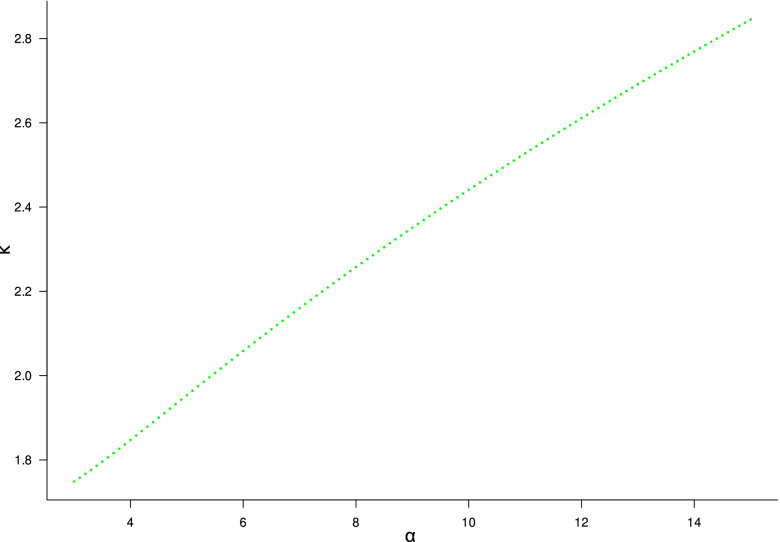
Relationship between α and *k* in the clinical range of α (3–15). In this case, $k=1.502+\left(0.092016\times \alpha \right)$, with *r*^2^ = 0.99, *p* < 0.001

Visual inspection of the relationship between *k* and α (in the clinical range of interest for α, *i.e.*, 3–15) shows a near-linear relationship (*r*^2^ = 0.99, *p* < 0.001, as depicted in Fig. 6), with $k=1.502+\left(0.092016\times \alpha \right)$.

Consequently, perfusion can be calculated from tissue enhancement as

$$\mathrm{Perfusion}=\frac{{y}_{\mathrm{max}}\times \left(\frac{1.502+\left(0.092016\times \alpha \right)}{{t}_{\mathrm{max}}}\right)}{\mathrm{Peak\;feeding\;vessel\;enhancement}}$$

and the tissue enhancement from perfusion as

$${y}_{\mathrm{max}}=\frac{\mathrm{Perfusion\;}\times \mathrm{\;Peak\;feeding\;vessel\;enhancement}}{\frac{(1.502+(0.092016\times \alpha )}{{t}_{\mathrm{max}}}}$$

## Discussion

Arterial phase tissue enhancement is usually clinically evaluated only by visual inspection as a semi-quantitative parameter. It is commonly assumed that to obtain quantitative data a full perfusion study is necessary, acquiring both the whole tissue enhancement curve and the feeding/outlet vessel enhancement curve through repeated scanning of the same anatomical region [7]. Therefore, while perfusion studies are performed for a wide spectrum of clinical applications [2, 14–16], they still imply the administration of large quantities of contrast agent, high image noise, and higher radiation dose from repeated scanning.

Organ perfusion can be computed with the deconvolution method [7], which requires the availability of the feeding/outlet vessel and tissue time-enhancement curves, and with the maximum slope method [8, 9]. The maximum slope method, proposed by Peters and Miles [8, 9], is based on the fact that the maximal rate of tissue contrast accumulation corresponds to the peak feeding vessel concentration. This method defines perfusion as the maximum slope of the time-enhancement curve divided by the peak enhancement of the feeding vessel. The two components of perfusion measurement are peak enhancement of the feeding vessel and tissue time-enhancement curve [17]. A bolus test can be used to estimate the peak enhancement of the feeding vessel. Likewise, the same bolus test can be used to estimate the tissue appropriate scan timing (*t*_max_, the peak of an arterial phase) if the volume of interest is included in the bolus test slice. As an example, if we are studying the coeliac circulation we can include both aorta and kidney to determine kidney and splanchnic circulation peak values [17]. The second component is the tissue time-enhancement curve. To determine the time-enhancement curve it has been always assumed that all data points need to be directly measured, scanning the same volume many times. We reasoned that, if the time-enhancement curve has a definite mathematical shape (gamma variate), it is possible to derive all data points using only the value of maximal enhancement obtained with just two CT scans (peak minus baseline) and then interpolate all the remaining data points using the curve shape parameters determined with the bolus test. We chose the Madsen formulation of the equation as it was simpler to handle [11]. The Madsen formulation of the gamma variate function expresses the time-enhancement curve as a function of its maximum value (*y*_max_), of the time at which the maximum is reached (*t*_max_), and of bolus shape (α). If these quantities are known, the maximal upslope can be computed and, together with the peak enhancement of the feeding vessel, used to compute tissue perfusion. Inspection of the gamma variate function or of patient tissue-enhancement curves shows that tissue enhancement reaches a plateau, which is maintained. Consequently, *y*_max_ does not need to be determined at a definite timepoint but can be measured in a range spanning several seconds: in most cases, the clinically acquired arterial phase falls into this timeframe. Bolus shape is described by the α parameter, which indicates the steepness of the bolus and contributes less than the other parameters (about 20%) in the determination of tissue perfusion. It is possible to estimate α from the bolus shape or, due to its relatively low contribution, use a fixed value which would describe aortic boluses in most patients with a normal cardiac output.

We retrospectively tested this theoretical framework on data obtained from abdominal perfusion studies performed in patients with advanced HCC who were candidate to anti-angiogenic therapy [3]. Time-enhancement curves were computed from ROIs on the HCC, the kidney cortex and pancreas, while a further ROI on the abdominal aorta allowed to compute its peak enhancement value and bolus shape (α). The time-enhancement curves were used to compute the maximum slope two times. The first computation was performed using all data points, smoothed with a spline function. The second computation was done by using just two values, one from the baseline CT scan and the other from the scan at *t*_max_: we calculated the maximum slope according to the Madsen formulation of the gamma variate using the α value of the bolus measured with the ROI in the abdominal aorta.

Linear regression and Bland–Altman analysis showed that the two perfusion computations gave similar values and were linearly related. The computation of maximum slope from a series of CT scans is not a direct measurement but a computation affected by sampling time and noise. Signal noise is usually removed with signal filtering, which corresponds to the removal of the highest frequencies. The highest frequencies correspond to the steepest part of the signal itself, reducing the absolute value of maximum slope computed from the curve. Likewise, a low time resolution of CT scan acquisitions does not allow to capture the maximum slope and leads to underestimation of the perfusion values: for example, in the first published application by Miles [9], a 2 mL/min/mL perfusion for the kidney cortex was reported, compared to an expected value of at least 5 mL/min/mL. Moreover, the maximum slope is mathematically a tangent, and a few degrees change in slope leads to large changes in absolute perfusion values: for example an angle increase of 10% (from 75 to 82.5°) leads to a tenfold increase of tangent/maximal slope of near 100% (from 3.73 to 7.59).

A similar approach has been proposed by Molloi and coworkers [18–20]. The authors used the Mullani-Gould method [21], which is a direct application of the Fick principle: if there is no venous outflow, perfusion is equal to tissue contrast concentration divided by the integral of the contrast concentration in the feeding vessel measured at the same time. The authors reasoned that the assumption of no venous outflow is valid with large volumes of interest (such as the entire perfusion bed of an artery or of a major arterial branch). They were therefore able to compute myocardial and lung perfusion by determining the whole amount of contrast entering the compartment of interest and the contrast concentration in the feeding vessel, using a baseline CT scan and a CT scan at peak enhancement time [18–20]. In any case, the computation of quantitative perfusion values must also reckon with the effects of different contrast flow rates. The assumption of correspondence between peak tissue enhancement and peak feeding vessel enhancement, as in the maximum slope method, holds true if there is no contrast outflow when peak tissue enhancement is measured, otherwise perfusion would be underestimated: a compact bolus obtained with a higher flow rate is less likely to meet this condition compared to a lower flow rate [22]. The 5 mL/s flow rate employed in this study is higher than the standard flow rate used in clinical studies (approximately 3.5 mL/s), while the amount of contrast used in clinical studies is usually higher, implying longer transit times and possible underestimation of computed perfusion values. Of note, while bolus length may also be prolonged in patients with diminished cardiac output (*e.g.*, patients with heart failure), the possible systematic underestimation would have no effect on the relationship between perfusion ratios and peak enhancement ratios but will need to be investigated in specific studies.

This proof-of-concept study has several limitations. First, its relatively small sample size, with patients retrospectively selected from a cohort of patients with advanced HCC. Second, acknowledging that the effect of respiratory oscillations might strongly influence partial volume effects, especially in thin tissues (such as the renal cortex), we tried to counter this phenomenon by drawing small ROIs. Third, all measurements were performed by a single reader. Considering the application of our model, the main limitation is the assumption of near simultaneous enhancement of all tissue included in the CT scan. We believe that this is true in the case of terminal circulation like the coeliac one or in the healthy brain, since arterial blood flow velocity is greater than 10 cm/s, but this assumption cannot be maintained in cases of significant arterial stenosis or when collateral circulation is present in the tissue of interest, delaying the peak tissue enhancement time, as it may happen in perfusion studies of patients with stroke. In this case, two CT scans would be able to detect a perfusion abnormality, but they would not allow to quantify it, as the presence of collateral circulation would result in delayed enhancement. Of note, absolute perfusion values are not mandatorily acquired in clinical applications, since, for example, the perfusion of HCC can be compared with absolute reference values from normal liver tissue or kidney cortex. Further limitations of our study are that we determined the aortic time-enhancement curves not using an actual bolus test but from the same data of the perfusion study and that peak tissue enhancement time was obtained from the gamma variate fitting and not actually subtracting only peak and baseline CT scans. We do not believe that this limits the general applicability of our results: indeed, the behaviour of time-enhancement curve has been described in detail in literature [17] and gamma fitting was employed to reduce the high noise inherent to perfusion study images.

In conclusion, we explored the relationship between organ perfusion and tissue enhancement measured on an arterial phase CT scan showing that (1) in a given tissue area, at peak enhancement time, the peak tissue enhancement is a definite computable function of perfusion, peak enhancement of the feeding vessel, and bolus shape; (2) the ratio of tissue enhancement in the arterial phase of two ROIs corresponds to the ratio of their perfusion values; (3) perfusion computed using only two frames of a time-enhancement curve was well related with the perfusion values computed using all the time enhancement curve, as the mathematical extrapolation of the time-enhancement curve could be superimposed to the measured data points.

## Supplementary Information

**Additional file 1.**

## Data Availability

Patients analysed in this study were previously reported in a manuscript (https://doi.org/10.1016/j.ejrad.2018.07.012) solely focused on the analysis of perfusion values obtained with the standard whole time-enhancement curve method. All data specifically generated for this study are reported in the manuscript or in its supplementary material.

## Abbreviations

CNR: Contrast-to-noise ratio
CT: Computed tomography
CTPI: Computed tomography perfusion imaging
HCC: Hepatocellular carcinoma
ROI: Region of interest
SNR: Signal-to-noise ratio
