# Supplementary material for: Computation of contrast-enhanced perfusion using only two CT scan phases: a proof-of-concept study on abdominal organs
Source: Eur Radiol Exp. 2022 Aug 29;6:37. doi: 10.1186/s41747-022-00292-y (PMC9420683; doi:10.1186/s41747-022-00292-y)
Supplement: Supplementary file 1 — Additional file 1. [file 41747_2022_292_MOESM1_ESM.pdf]

ELECTRONIC SUPPLEMENTARY MATERIAL

Computation of contrast-enhanced perfusion using only two CT scan phases: a proof-of-concept study on abdominal organs

The special case of the lung

As in ventilation-perfusion scintigraphy, it is possible to express lung perfusion as fraction of cardiac output, since all output from the right ventricle is delivered to lungs, which can be divided in an arbitrary number of ROIs:

$$Total\ lung\ perfusion = Cardiac\ output = \sum_{k=1}^{n} Perfusion_{ROI_k}$$

and, as previously seen,

$$Total\ lung\ perfusion = Cardiac\ output = \sum_{k=1}^{n} \frac{Maximum\ slope\ of\ tissue\ enhancement_{ROI_k}}{Peak\ feeding\ vessel\ enhancement_{ROI_k}}$$

from which we can derive

$$1 = \sum_{k=1}^{n} \frac{Maximum\ slope\ of\ tissue\ enhancement_{ROI_k}}{Peak\ feeding\ vessel\ enhancement_{ROI_k}} \times \frac{1}{Total\ lung\ perfusion}$$

or

$$1 = \frac{\sum_{k=1}^{n} Maximum\ slope\ of\ tissue\ enhancement_{ROI_k}}{Total\ lung\ perfusion \times Peak\ feeding\ vessel\ enhancement_{ROI_k}}$$

The contribution of each ROI to the total is therefore the same regardless of the denominator, which includes both the total perfusion and the peak inlet vessel enhancement. Consequently, the contribution of each ROI is only function of its maximum slope, as the peak inlet vessel enhancement acts as denominator:

$$Perfusion_{ROI_1} = \frac{Maximum\ slope\ of\ tissue\ enhancement_{ROI_1}}{\sum_{k=1}^{n} Maximum\ slope\ of\ tissue\ enhancement_{ROI_k}}$$

As described before, the maximum slope of tissue enhancement in a ROI is defined as the ROI maximum enhancement (y_{max}) divided by a constant k, that can be therefore included in the denominator with both the peak enhancement of the inlet vessel and the total enhancement.

Lastly, if y_{max} is not measured at peak but during the ascending part of the enhancement curve, the measured enhancement will be proportional to the true y_{max} by a constant k_2, as:

$$Measured\ y_{max} = peak\ y_{max} \times k_2$$

and k_2 can be grouped with k and peak enhancement of the inlet vessel.

Application workflow

Our model to assess organ perfusion would then call for eight steps to be performed, as follows:

1. A bolus test is performed injecting a small bolus of iodinated contrast (*i.e.*, 5–10 ml) and acquiring a single-slice low-dose scan similar to the one used for bolus tracking, including the inlet vessel (*e.g.*, the abdominal aorta for coeliac/mesenterial circulation) and a well-perfused region of the vascular territory which we want to study (e.g., on the kidney for coeliac/mesenterial circulation). In parenchymatous organs, $t_{max\text{-}tissue}$ corresponds to the bolus arrival time plus injection length [1] while, if the contrast is completely intravascular (as in the brain) corresponds to the peak of bolus. Two ROIs are drawn: the first on the inlet vessel and the second on the well-perfused region.
2. The values of y_{max}, t_{max}, and α are determined on the aortic enhancement curve. The $t_{max\text{-}tissue}$ of the territory of interest is computed from tissue ROI.
3. The aortic curve is corrected computing the expected enhancement with the contrast dose needed to perform the clinical study, according to contrast concentration/enhancement curves [1].
4. An arterial phase CT scan is performed at $t_{max\text{-}tissue}$ of the tissue in study.
5. Arterial phase tissue enhancement (measured in Hounsfield units [HU]) is computed by subtracting the enhancement in the pre-contrast acquisition from the enhancement in the post-contrast acquisition. For highly perfused structures, pre-contrast enhancement can be assumed to be close to zero and not directly measured.

6. The maximum slope of the tissue curve is computed as the maximum of the first derivative of gamma variate function divided by t_{max} in order to scale it and obtain the peak enhancement in HU/s, as:

$$dy = y_{max} \times \left(\left(\alpha \times t^{\alpha-1} \times e^{\alpha(1-t)} \right) - \left(\alpha \times t^{\alpha} \times e^{\alpha(1-t)} \right) \right)$$

where we use as *t* the zero of the second derivative, expressed as $t = \frac{(\alpha \pm \sqrt{\alpha})}{\alpha}$

7. ROI perfusion is computed dividing the maximum slope of the tissue curve (point 6) by the peak inlet vessel enhancement.

References

1. Bae KT (2010) Intravenous Contrast Medium Administration and Scan Timing at CT: Considerations and Approaches. Radiology 256:32–61. doi:10.1148/radiol.10090908.
